# CCL14 serves as a novel prognostic factor and tumor suppressor of HCC by modulating cell cycle and promoting apoptosis

**DOI:** 10.1038/s41419-019-1966-6

**Published:** 2019-10-22

**Authors:** Mengxuan Zhu, Weiyue Xu, Chuanyuan Wei, Jing Huang, Jietian Xu, Yuye Zhang, Yan Zhao, Jie Chen, Shuangshuang Dong, Binbin Liu, Chunmin Liang

**Affiliations:** 1 Liver Cancer Institute, Zhongshan Hospital, Fudan University, Key Laboratory of Carcinogenesis and Cancer Invasion, Ministry of Education, Shanghai, P.R. China; 2 Laboratory of Tumor Immunology, Department of Anatomy, Histology, and Embryology, School of Basic Medical Sciences, Fudan University, Shanghai, P.R. China

**Keywords:** Cancer prevention, Diagnostic markers

## Abstract

CCL14 is a member of CC chemokines and its role in hepatocellular carcinoma (HCC) is still unknown. In this study, CCL14 expression were analyzed by tissue microarray (TMA) including 171 paired tumor and peritumor tissues of patients from Zhongshan Hospital of Fudan University. We found for the first time that CCL14 was downregulated in HCC tumor tissues compared with peritumor tissues (*P* = 0.01). Meanwhile, CCL14 low expression in HCC tumor tissues is associated with a poor prognosis (*P* = 0.035). CCL14 also displayed its predictive value in high differentiation (*P* = 0.026), liver cirrhosis (*P* = 0.003), and no tumor capsule (*P* = 0.024) subgroups. The underlying mechanisms were further investigated in HCC cell lines by CCL14 overexpression and knock-down in vitro. We found overexpression of CCL14 suppressed proliferation and promoted apoptosis of HCC cells. Finally, the effect was confirmed by animal xenograft tumor models in vivo. The results shown overexpression of CCL14 lead to inhibiting the growth of tumor in nude mice. Interestingly, our data also implied that CCL14 played these effects by inhibiting the activation of Wnt/β-catenin pathway. These findings suggest CCL14 is a novel prognostic factor of HCC and serve as a tumor suppressor.

## Introduction

Hepatocellular carcinoma (HCC), one of the most common malignant tumors worldwide, ranks fifth in incidence and second in cancer-related mortality^1^. The prognosis of HCC remains poor due to its insidious onset and high rate of recurrence after resection. Over the past few years, molecular targeted therapy has been proven to be effective in patients. However, targeted therapy is limited because drug resistance is easily developed. Thus, it is urgent to develop new treatments for HCC.

Chemokines were initially defined as molecular signals to induce leukocyte migration during inflammation, which have been identified and classified into four subfamilies (CXC, CC, CX3C, and C)^2^. Recently, researchers have demonstrated that CCL2 and CCL5 promote cancer cell proliferation, invasion and metastasis in several tumors^3–7^.

Consistent with other reports, we have focused on chemokines for years and previously found that secondary lymphoid tissue chemokine (SLC, also named as CCL21) can attract dendritic cells (DCs) to T cells, serving as a treatment for tumor^8–11^. Moreover, it was also reported that CC chemokine receptor-like 1 play its function as tumor suppressor, which inhibits the proliferation and invasion of HCC cells^12^.

In this study, we aim to assess the function of CCL14 in HCC. We found low expression of CCL14 in HCC tumor tissues lead to shorter OS. Overexpression of CCL14 by lentivirus can suppress the proliferation and promote the apoptosis of HCC cells, which lead to inhibiting tumor growth in animal xenograft tumor models. Our results also suggest that CCL14 may play these effects by inhibiting the activation of Wnt/β-catenin pathway.

## Materials and methods

### Clinical samples

Tissue microarray (TMA), including 171 paired tumor and peritumor tissues, was obtained from Zhongshan Hospital, Fudan University (Shanghai, China). All patients were confirmed as HCC by two pathologists independently and underwent complete surgical excision between January 2006 and August 2006. Another 20 pairs of fresh tumor and peritumor tissues were randomly collected from the Liver Cancer Institute at Zhongshan Hospital and analyzed by western blot. This study was approved by the ethics committee of Zhongshan Hospital and informed consent was obtained from each patient.

### Immunohistochemistry (IHC)

IHC was performed in TMA as previously described^13,14^. The immunostaining intensities were semiquantitatively scored as: 0 for negative; 1 for weak; 2 for moderate; 3 for strong by two observers independently. Groups 0 and 1 were classified as low expression, while groups 2 and 3 were classified as high expression.

### Cell culture

The HCC cell lines Huh7, SMMC-7721, and HepG2 were purchased from Chinese Academy of Sciences (Cell Bank, Shanghai Institutes for Biological Sciences). The stepwise metastatic HCC cell lines including MHCC97L, MHCC97H, and HCCLM3 were established by the Liver Cancer Institute of Zhongshan Hospital. All cell lines were routinely cultured.

### Western blot assay

Western blot was performed as previously described^15^. ECL system (NCM Biotech) was used to visualize the signals. GAPDH was used as a loading control.

### Enzyme-linked immunosorbent assay (ELISA)

To detect the secreted CCL14 levels, cell culture supernatants were harvested after 48 h and analyzed using HCC-1/CCL14 Human ELISA Kit (Raybiotech) according to the manufacturer’s protocols.

### CCL14 overexpression and downregulation

Stable over-expression of CCL14 in LM3 was constructed by recombinant Lentivirus encoding CCL14. Blank lentivirus was used as vector control. The knockdown of CCL14 in Huh7 cells was constructed by transfecting specific siRNA with Lipofectamine 2000 (Invitrogen) according to manufacturer’s instructions. The siRNA target sequences were as follows: ***siRNA-1***: CCAUCGCCCUAGGGACCAATT; ***siRNA-2***: CCAACAGCC AGUGCUCCAATT.

### CCK-8 colony formation assay and BrdU assay

The CCK8 assay was performed as previously described^16^. BrdU assay was performed according to the manufacturer’s instructions.

### Flow cytometric analysis

Flow cytometric analysis was used to detect cell cycle and apoptosis as described previously^17^. Apoptosis was measured using FITC-AnnexinV Apoptosis Detection system (BD Bioscience) according to the manufacturer’s instructions.

### Tumor bearing animal models in nude mice

LM3 or Huh7 cells with different CCL14 expression levels were harvested and suspended in serum-free DMEM. Athymic nude mice (4–6 weeks old) were divided into four groups: (1) CCL14 group (*n* = 5), each mouse injected into the lower back with 2 × 10^6^ CCL14 overexpressed LM3 cells; (2) Vector group(*n* = 5), each mouse injected with 2 × 10^6^ blank vector infected LM3 cells; (3) si-NC group (*n* = 5), each mouse injected into the lower back with 3 × 10^6^ Huh7 cells treated with siRNA control; (3) siRNA2 group (*n* = 5), each mouse injected into the lower back with 3 × 10^6^ Huh7 cells threated with CCL14 siRNA. Every 6 days, tumors were monitored and measured. At 42^nd^ day, the mice were sacrificed and tumors weight were examined.

### Statistical analysis

The results were expressed as the means ± SD and were considered to be statistically significant at *p* < 0.05. The data were analyzed using IBM SPSS Statistics 20 (IBM Corporation, USA). Student’s *t* test was used for comparisons between groups. Categorical data were analyzed by the chi-square or Fisher’s exact tests. Kaplan–Meier analyses and log-rank tests were used to perform survival analyses.

## Results

### CCL14 is downregulated in HCC tissues and CCL14 low expression is associated with a poor prognosis in clinical patients

**Fig. 1:**
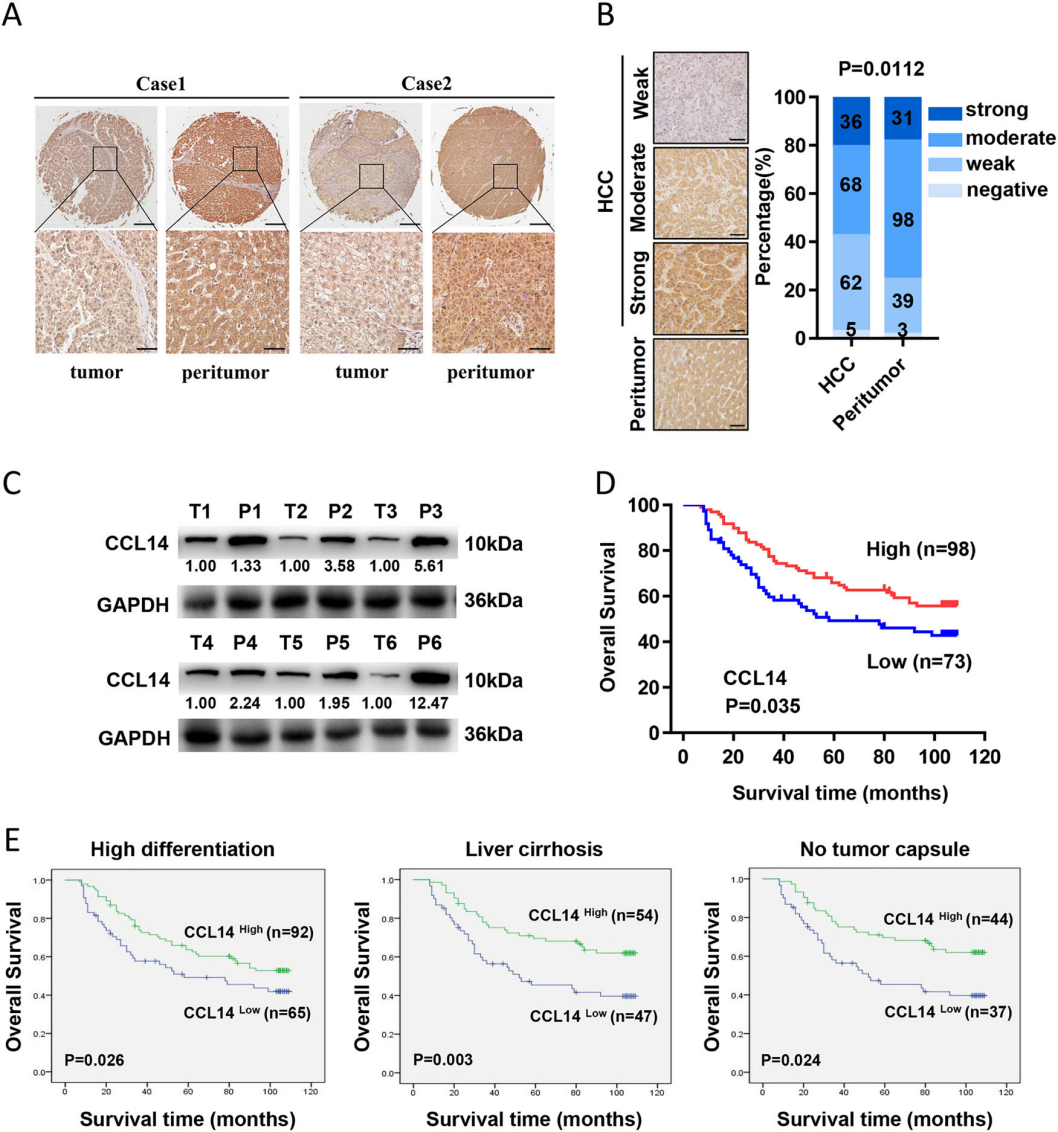
Relative CCL14 expression in HCC tissues and its clinical significance. **a** Representative images of TMA stained with IHC for CCL14. Scale bar, ×400, 50 μm. **b** The CCL14 protein expression of hepatocellular carcinoma and peritumor tissues in TMA was analyzed. **c** The CCL14 protein expression in representative six pairs of hepatocellular carcinoma tissues and representative bands are shown. **d** Kaplan–Meier curves for overall survival from 171 HCC patients according to CCL14 expression in TMA. **e** Prognostic value of CCL14 in high differentiation, liver cirrhosis, and no tumor capsule subgroups

CCL14 expression profile was accessed by IHC in a human TMA containing 171 paired HCC and peritumor tissues and representative images were shown in Fig. 1a. The results indicated that CCL14 were scored as negative or weak expression in 42.69% (73 of 171: negative, *n* = 5; weak, *n* = 68) of HCC tissues, as compared with 24.56% of corresponding peritumor tissues (42 of 171: negative, *n* = 3; weak, *n* = 39; *P* = 0.0112; Fig. 1b). Twenty pairs of frozen tissues from HCC patients were randomly chosen to examine CCL14 protein expression. Western blot analysis showed that the protein level of CCL14 was remarkably lower in HCC tissues than in adjacent tissues, as shown in representative six pairs (Fig. 1c).

All patients in the TMA were classified into the CCL14^low^ group or CCL14^high^ group according to CCL14 expression. The results showed CCL14 low expression was associated with a poorer prognosis by Kaplan–Meier analysis (Fig. 1d). The predictive value of CCL14 were also observed in high differentiation (*P* = 0.026), liver cirrhosis (*P* = 0.003) and no tumor capsule (*P* *=* 0.024) subgroups (Fig. 1e). Additionally, as shown in Table 1, low expression of CCL14 was also associated with microvascular invasion (*P* = 0.039).

**Table 1:** Association of CCL14 expression with clinical parameters of 171 HCC patients

Clinical characteristic	CCL14^Low^ no.	CCL14^High^ no.	*P*-value
*Age (no.)*			0.478
<60	54	74	
≥60	19	24	
*Gender*			0.268
Male	61	77	
Female	12	21	
*Tumor differentiation*			0.195
I–II	8	6	
III–IV	65	92	
*Tumor size (cm)*			0.217
<5	41	62	
≥5	32	36	
*T*umor number			0.201
Single	59	85	
Multiple	14	13	
*TNM stage*			0.172
I–II	62	89	
III–IV	11	9	
*BCLC stage*			
0–A	26	44	0.144
B–C	47	54	
*Microvascular invasion*			0.039*
No	41	69	
Yes	32	29	
*Tumor capsule*			0.276
No	37	44	
Yes	36	54	
*History of cirrhosis*			0.107
No	12	25	
Yes	61	73	

*Statistically significant

A significant association between the CCL14 expression signature and OS in the univariable Cox regression model was also observed. As shown in Table 2, the HR value of the CCL14 ^low^ group vs. the CCL14^high^ group for OS was 0.631 (*P* = 0.037). At the same time, tumor size (HR = 1.890; *P* = 0.004), microvascular invasion (HR = 1.971; *P* = 0.002), TNM stage (HR = 2.273; *P* = 0.004), and BCLC stage (HR = 1.839; *P* = 0.010) were contributing factors to a shorter OS of patients.

**Table 2:** Univariate and multivariate analysis of CCL14 in OS of 171 HCC patients

Variables		Univariate analysis			Multivariate analysis		
		HR	95%CI	*P*-value	HR	95%CI	*P*-value
Age,year	<60 vs. ≥60	1.561	0.988–2.465	0.056	NA		
Gender	Male vs. female	0.921	0.540–1.571	0.762	NA		
Tumor size (cm)	≤5 vs. >5	1.890	1.225–2.915	0.004*	1.749	0.891–3.432	0.104
Tumor number	Mutiple vs. single	1.682	0.986–2.870	0.056	NA		
Tumor differentiation	I–II vs. III–IV	2.109	0.772–5.760	0.146	NA		
Microvascular invasion	Yes vs. no	1.971	1.276–3.045	0.002*	1.899	1.039–3.471	0.037*
Tumor capsule	Yes vs. no	1.208	0.783–1.863	0.393	NA		
History of cirrhosis	Yes vs. no	1.155	0.698–1.912	0.576	NA		
TNM stage	I–II vs. III–IV	2.273	1.297–3.983	0.004*	1.522	0.802–2.890	0.199
BCLC stage	0–A vs. B–C	1.839	1.154–2.930	0.010*	0.747	0.323–1.727	0.495
CCL14	Low vs. high	0.631	0.409–0.973	0.037*	0.703	0.452–1.094	0.118

*Note:* Univariate and multivariate analyses, Cox proportional hazards regression model

*OS* overall survival time, *HR* hazard ratio, *CI* confidence interval, *NA* not applicable

*Statistically significant

### Overexpression of CCL14 inhibits the proliferation of HCC cells in vitro

**Fig. 2:**
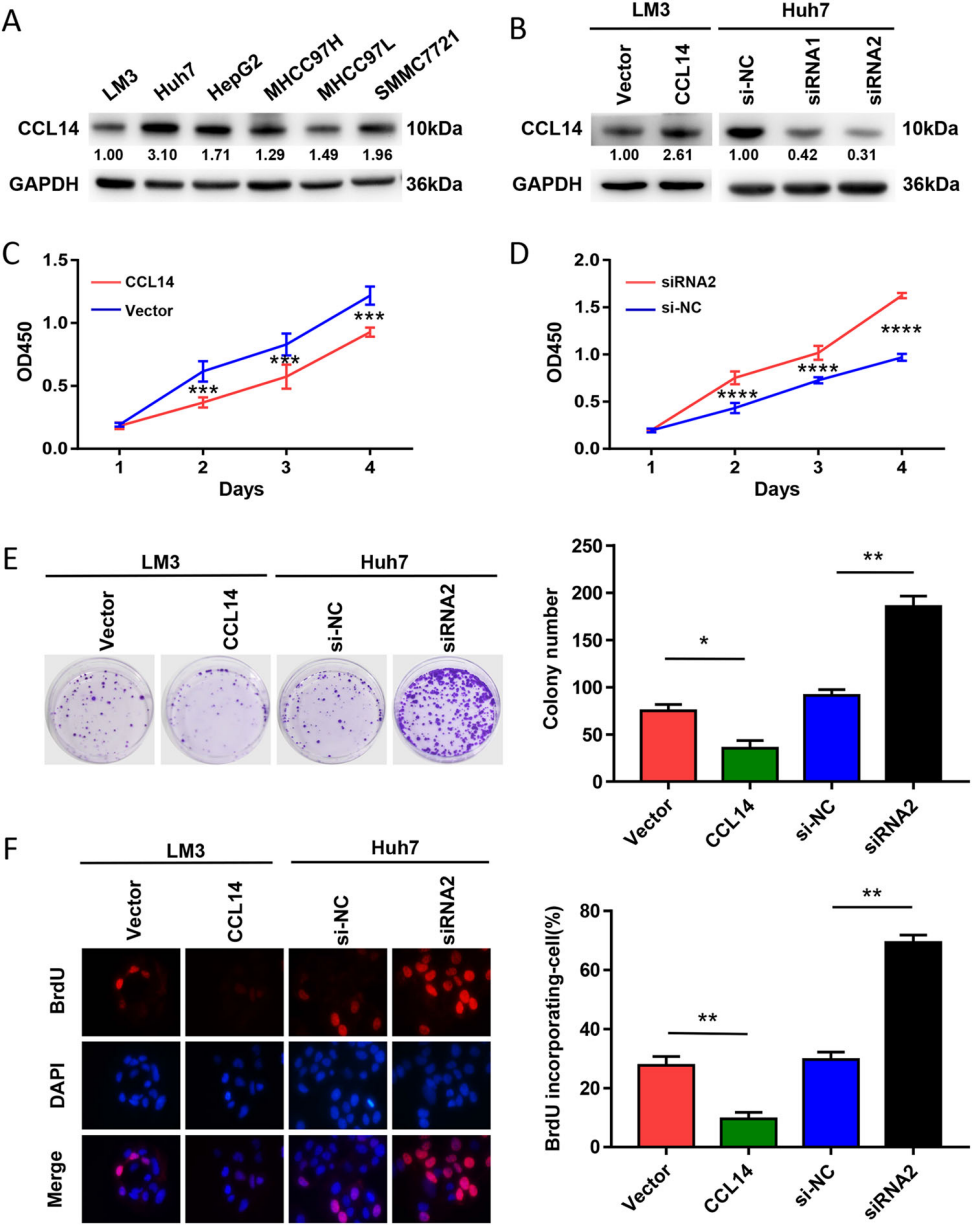
CCL14 inhibits the proliferation of HCC cells in vitro. **a** CCL14 were measured by Western blot in six HCC cell lines, including SMMC-7721, Huh-7, HepG2, Hep3B, MHCC-97L, MHCC-97H, and HCCLM3. **b** CCL14 was overexpressed in LM3 cells by recombinant lentivirus encoding CCL14 (CCL14 group) and knocked down by two siRNA-specific sequences targeting CCL14 in Huh7 cells (siRNA1, siRNA2 groups), which were confirmed by Western blot. siRNA2 sequences played more specific functions. Vector group means LM3 cells were infected by blank lentivirus as control group. si-NC group means Huh7 cells were infected by non-specific siRNA for CCL14 as normal control group. **c** Over-expression of CCL14 significantly reduced the growth rate of LM3 cells by CCK8 assays. **d** Down-regulation of CCL14 by siRNA significantly increased the growth rate in Huh7 cells by CCK8 assays. **e** Overexpression of CCL14 in LM3 cells increased the bacterial colonies, whereas downregulation of CCL14 in Huh7 cells decreased the bacterial colonies. **f** Representative micrographs and quantification of BrdU incorporation in the indicated cells. *P < 0.05, **P < 0.01, ***P < 0.001, ****P < 0.0001

The expression of CCL14 in different human HCC cell lines were tested by western blot, including SMMC-7721, Huh-7, HepG2, Hep3B, MHCC-97L, MHCC-97H, and HCCLM3. It revealed that CCL14 was highly expressed in Huh-7 cells while lowly expressed in LM3 cells (Fig. 2a). CCL14 expression and secretion were both successfully enhanced by recombinant lentivirus in LM3 cells, whereas knocked down in Huh7 cells by siRNA (Fig. 2b, Supplementary Fig. 1). Because siRNA2 sequences played more specific functions, we choose siRNA2 for the following tests. Over-expression of CCL14 significantly reduced the growth rate of LM3 cells, which was revealed by CCK8 assays (Fig. 2c). On the contrary, down-regulation of CCL14 by siRNA2 significantly increased the growth rate in Huh7 cells (Fig. 2d). At the same time, the over-expression of CCL14 in LM3 cells decreased the colonies, while downregulation of CCL14 in Huh7 cells increased the colonies (Fig. 2e). Additionally, CCL14 overexpression in LM3 decreased the proportion of BrdU-incorporated cells, while CCL14 knockdown in Huh7 increased the proportion of BrdU-incorporated cells (Fig. 2f).

### CCL14 modulates cell cycle and promotes apoptosis in vitro

**Fig. 3:**
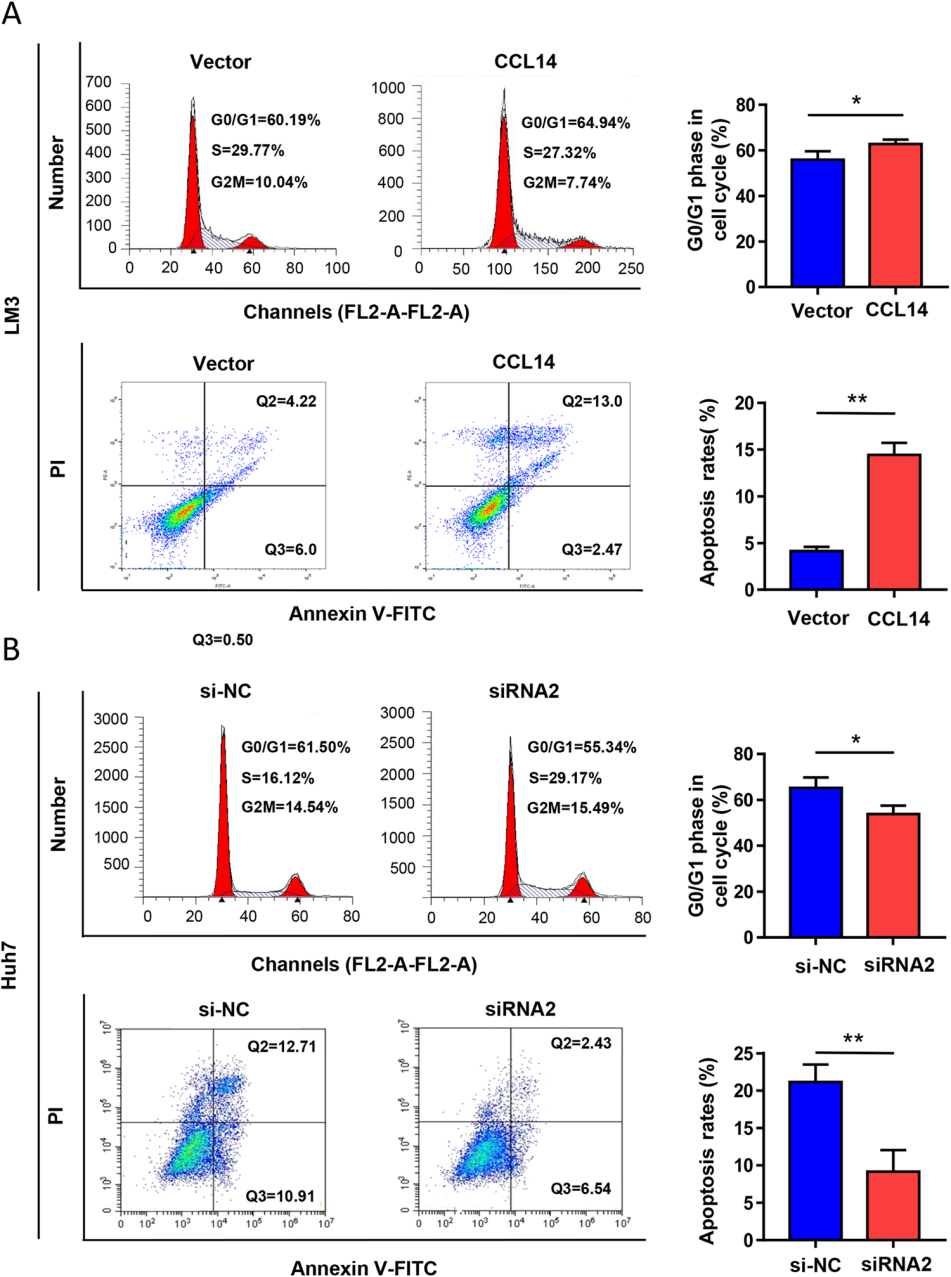
CCL14 modulates cell cycle and promotes apoptosis. **a** Overexpression of CCL14 in LM3 increased the percentage of G0/G1 phase cells and the rate of apoptosis cells by flow cytometry analysis. **b** Knock-down of CCL14 in Huh7 decreased the percentage of G0/G1 phase cells and the rate of apoptosis cell by flow cytometry analysis. *P *<* 0.05, **P < 0.01

CCL14-mediated changes in cell cycle progression and apoptosis were accessed by flow cytometry analysis. Overexpression of CCL14 in LM3 increased both the percentage of G0/G1 phase cells and the rate of apoptosis cells (Fig. 3a, *P < 0.05, **P < 0.01). Knock-down of CCL14 in Huh7 decreased the percentage of G0/G1 phase cells and the rate of apoptosis cell (Fig. 3b, *P *<* 0.05, **P < 0.01). These data indicated that CCL14 suppressed HCC cell proliferation by inhibiting cell cycle progression and promoting apoptosis in HCC cells.

### Overexpression of CCL14 inhibits the tumor growth in nude mice in vivo

**Fig. 4:**
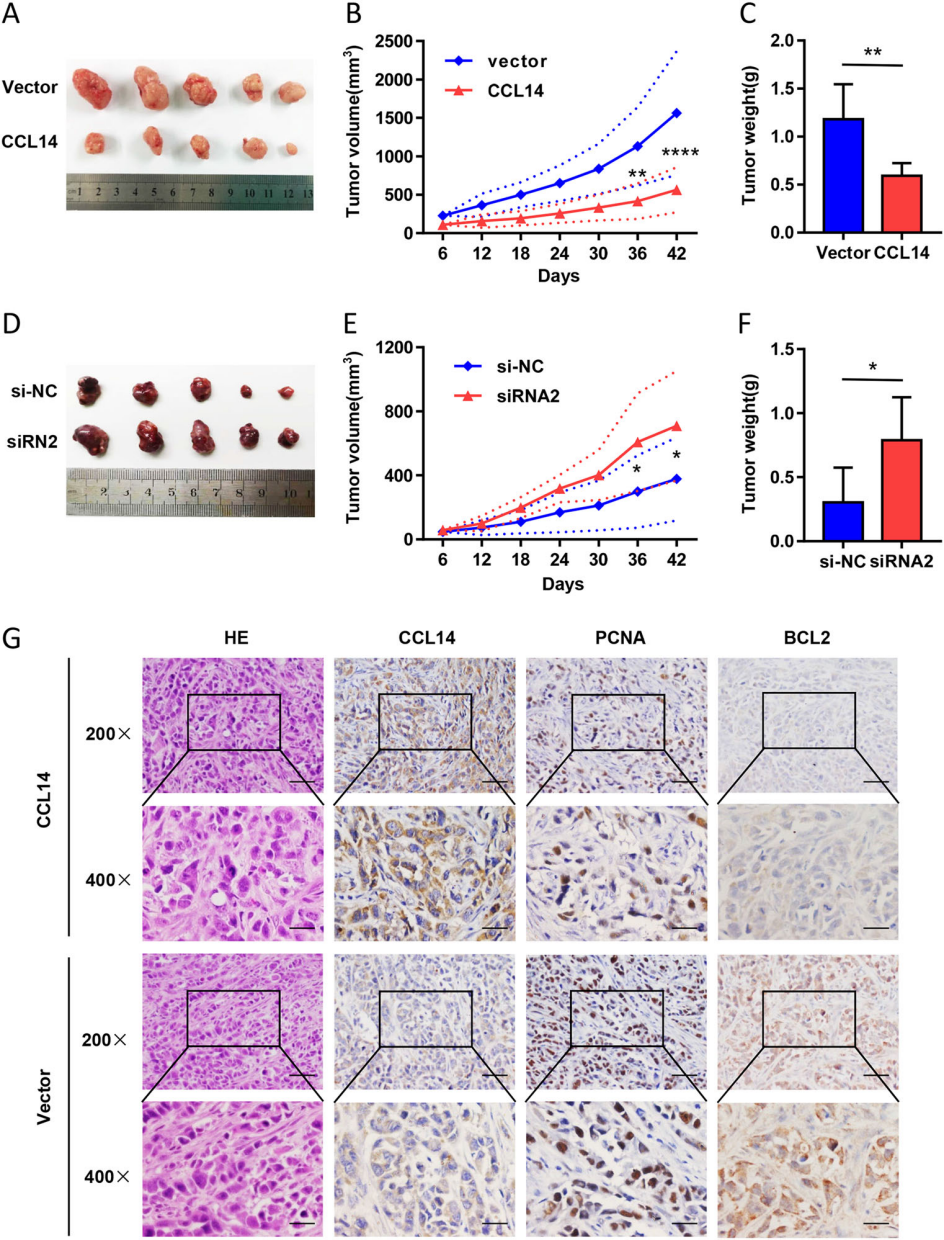
CCL14 inhibits tumor growth of HCC in nude mice in vivo. **a** Images of excised tumors from five nude mice at 42 days. Compared to control vector group, tumors were smaller in CCL14 group. **b** Tumor-bearing animal models (*n* = 5) were constructed. The tumor volumes were measured every 6 days. Tumor grew slowly in CCL14 group than control vector group. **c** The tumor weight was decreased in CCL14 group. **d** Images of si-NC group and siRNA2 group tumors at 42 days. Compared to si-NC group, tumors were bigger in siRNA2 group. **e** Tumor grew faster in siRNA2 group than si-NC group. **f** The tumor weight was increased in siRNA2 group. **g** Representative graphs of H&E staining and immunohistochemical staining for PCNA and BCL2 in sections. Tumor sections of CCL14 group displayed lower PCNA and BCL2 staining. **P < 0.01, ***P < 0.001, ****P < 0.0001

In tumor-bearing animal models, we found tumor grew slowly in CCL14-overexpressed group than control group (Fig. 4a). Both tumor volume and weight were decreased at the same time (Fig. 4b, c). Consistently, tumors of siRNA2 group grew faster and bigger than si-NC group (Fig. 4d–f). Moreover, IHC analysis confirmed that CCL14 over-expressed tumors displayed lower PCNA and BCL2 staining (Fig. 4g). Our results indicate that CCL14 suppresses HCC growth in vivo.

### CCL14 inhibits the proliferation of HCC cells via Wnt/β-catenin-signaling pathway

**Fig. 5:**
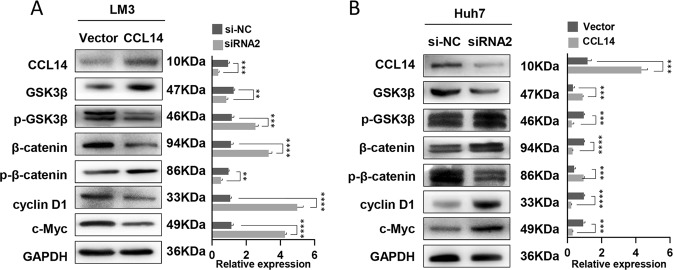
Regulation of Wnt/β-catenin signaling pathway by CCL14. **a** Several key proteins of the Wnt/β-catenin-signaling pathway were analyzed by western blot in LM3 cells. Over-expression of CCL14 by Lentivirus decreased the expression of p-GSK3β (S9), β-catenin, cyclin D1, and c-Myc. **b** Western blot analyses were also performed to test Wnt/β-catenin-signaling pathway in Huh7 cells. Down-regulation of CCL14 by siRNA2 significantly increased the expression of p-GSK3β (S9), β-catenin, cyclin D1, and c-Myc. **c** Representative images of immunohistochemical staining for cyclinD1 and c-Myc in TMA. CCL14 expression was negatively correlated with cyclinD1 and c-Myc levels. **P < 0.01, ***P *<* 0.001, ***P *<* 0.001

The expression of several key proteins of Wnt/β-catenin pathway in HCC cells were analyzed. Overexpression of CCL14 decreased the expression of p-GSK3β (S9), β-catenin, cyclin D1, and c-Myc in LM3 cells (Fig. 5a). On the contrary, down-regulation of CCL14 by siRNA significantly increased the expression of these proteins in Huh7 cells (Fig. 5b). In HCC samples, CCL14 expression also showed negatively correlated with cyclin D1 levels (as shown in Supplementary Fig. 2).

**Fig. 6:**
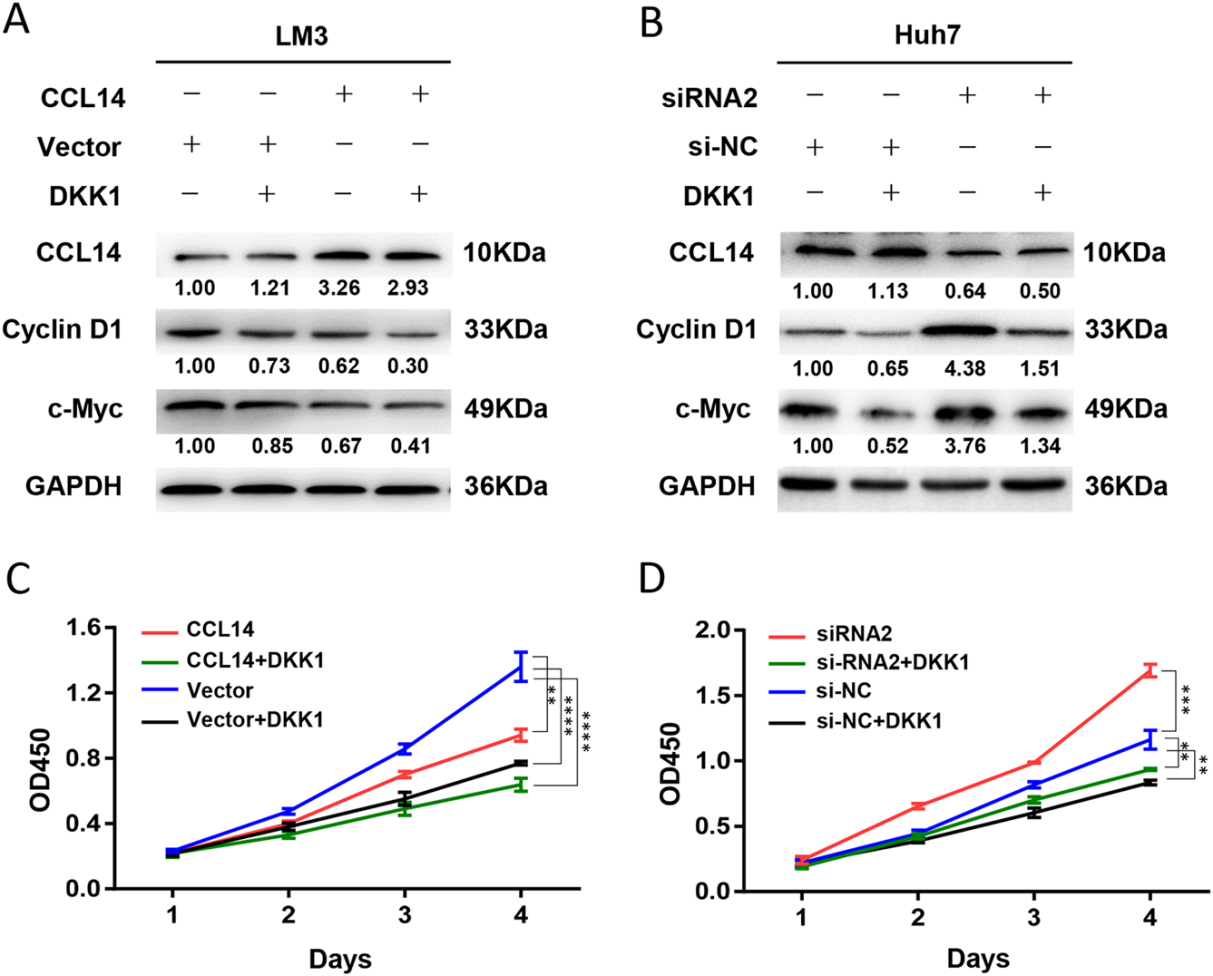
CCL14 inhibited proliferation of HCC cells via Wnt/β-catenin-signaling pathway. **a** LM3 cells were transfected with control vector and CCL14-lentivirus, treated with DKK1 and harvested for Western blot assay. **b** Huh7 cells transfected with si-NC and siRNA2, treated with DKK1 and harvested for Western blot assay. **c** LM3 cells were transfected with control vector and CCL14-lentivirus, treated with DKK1 and detected by CCK8 assay. **d** Huh7 cells transfected with si-NC and siRNA2, treated with DKK1 and detected by CCK8 assay. **P < 0.01, ***P *<* 0.001, ****P < 0.0001

Dickkopf1 (DKK1), the specific inhibitor of Wnt/β-catenin-signaling pathway was used to investigate whether CCL14 inhibits the proliferation of tumor cells via Wnt/β-catenin pathway. In LM3 cells, as shown in Fig. 6a, c, compared with blank-vector control, over-expression of CCL14 decreased the expression of cyclinD1 and c-Myc, at the same time, significantly inhibited the proliferation of LM3 cells (**P *<* 0.01). The combination of CCL14 and DKK1 caused highest inhibition effect (****P *<* 0.0001).

On the other hand, in Huh7 cells, as shown in Fig. 6b, d, compared with si-NC control transfection, CCL14 knocking-down by siRNA2 transfection lead to an increase of cyclinD1 and c-Myc, as well as the increased proliferation (***P *<* 0.001). The combination of DKK1 played an opposite inhibition effect (**P *<* 0.01).

Additionally, we also explored the potential mechanisms that may lead to CCL14 downregulation in HCC and found that CCL14 levels can be restored by DNA demethylation agent 5-Aza-CdR and histone demethylase inhibitor GSK-467 (Supplementary Fig. 3), indicating the low level of CCL14 is partially the result of epigenetic regulation.

## Discussion

CCL14 induces monocytes, macrophages, and THP-1 cells by binding to CCR1, CCR3, and CCR5^18^. Previous reports have shown that CCL14 is also involved in the pathogenesis and progression of various disorders, including allergic airway inflammation and some cancers^19–21^.

In this study, we examined the expression of CCL14 in paired HCC tumor tissue and peritumor tissues. The results showed that CCL14 low expression in HCC tumor tissues was correlated with poor survival. We also found overexpression of CCL14 inhibited proliferation and promoted the apoptosis of LM3 cells. Knock-down of CCL14 showed opposite effects in Huh7 cells. Moreover, CCL14 suppressed tumor growth in animal models. So CCL14 suppresses the progression and promotes the apoptosis of HCC cells, which contribute to longer OS in HCC patients. In addition, consistent with previous research that reported CCL14 expression was suppressed by JARID1B/LSD1/NuRD complex in breast cancer^21^, we found CCL14 expression was upregulated after being treated with DNA demethylation agent 5-Aza-CdR or histone demethylase inhibitor GSK-467. These results suggested that epigenetic regulation play critical role in the expression of CCL14 and we will explore more detailed mechanisms subsequently.

The Wnt/β-catenin pathway has been found in various cancer types, including HCC^22–24^. Previous studies have reported that β-catenin plays an important role in Wnt/β-catenin signaling. When Wnt proteins bind to the receptor, β-catenin will translocate to the nucleus and interact with TCF/LEF transcription factors to regulate downstream gene expression^25^. In the present study, we showed that knocking-down CCL14 could increase p-GSK3β (S9), β-catenin (S33/S37), and further promote the expression of c-myc and cyclin D1, which are the downstream target genes of Wnt/β-catenin and associated with tumor cell proliferation or apoptosis in various cancers^26–28^. Our results also suggested that CCL14 exerts its regulatory effects on proliferation of HCC cells through the Wnt/β-catenin pathway by the treatment of DKK1, the specific inhibitor of Wnt/β-catenin pathways.

These findings suggest CCL14 is a novel prognostic factor of HCC and serve as a tumor suppressor.

## Supplementary information

Supplementary Figure 1

Supplementary Figure 2

Supplementary Figure 3

Figure Legends for Supplementary Figures
