# Supplementary figures and images for: CCL14 serves as a novel prognostic factor and tumor suppressor of HCC by modulating cell cycle and promoting apoptosis
Source: Cell Death Dis. 2019 Oct 22;10(11):796. doi: 10.1038/s41419-019-1966-6 (PMC6805940; doi:10.1038/s41419-019-1966-6)

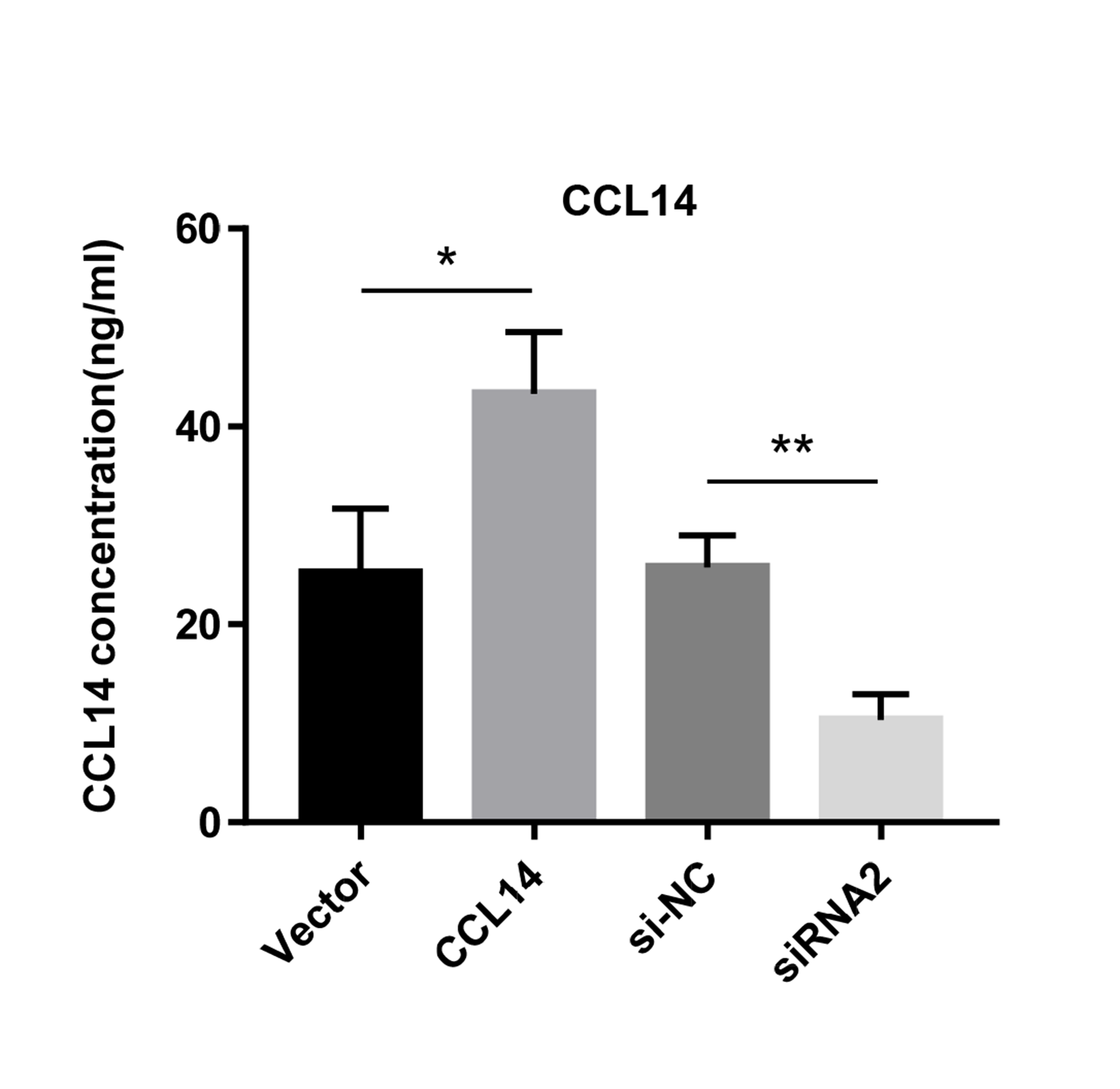

Supplement: Supplementary file 1 — Supplementary Figure 1 [file 41419_2019_1966_MOESM1_ESM.tif]

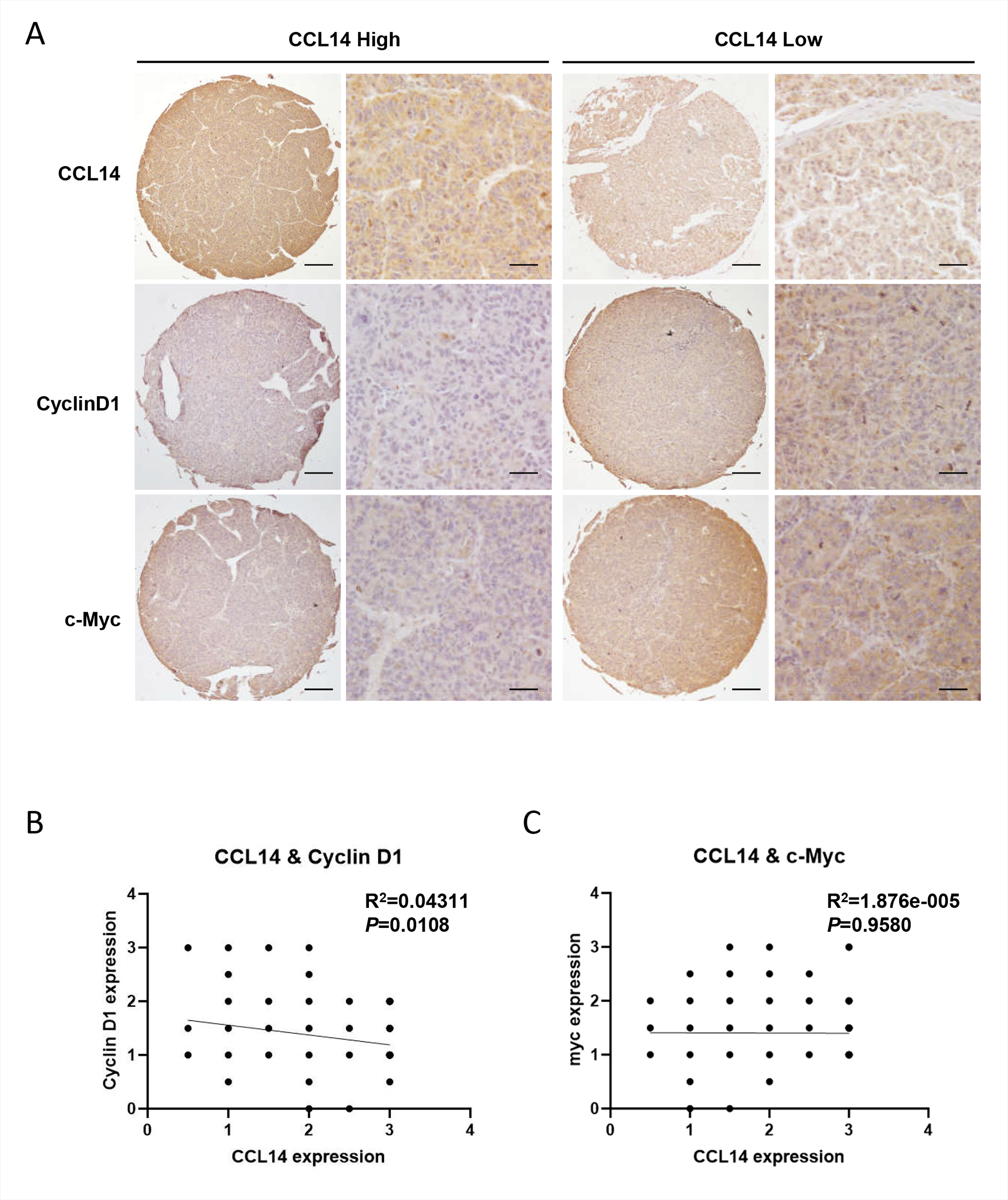

Supplement: Supplementary file 2 — Supplementary Figure 2 [file 41419_2019_1966_MOESM2_ESM.tif]

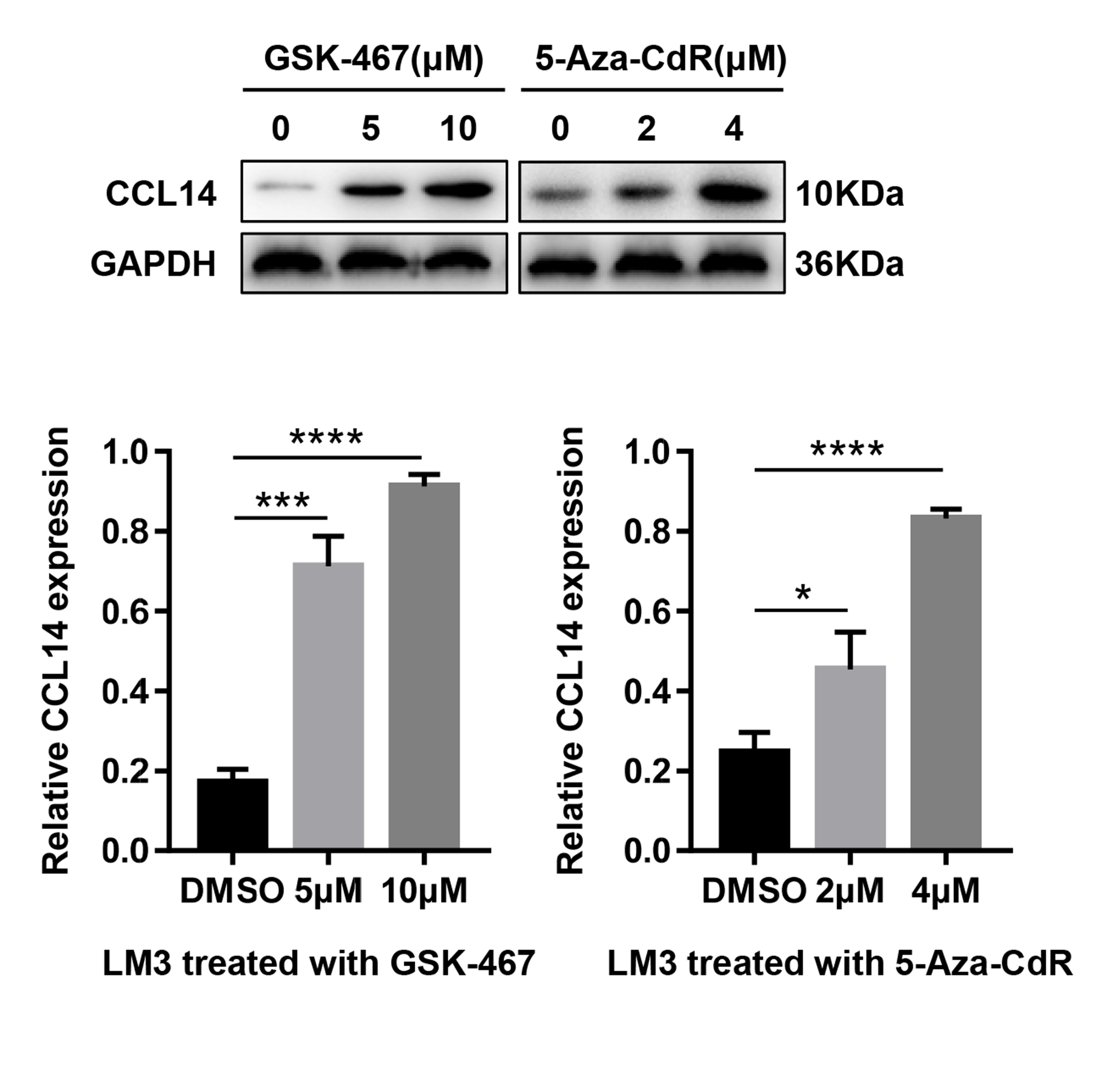

Supplement: Supplementary file 3 — Supplementary Figure 3 [file 41419_2019_1966_MOESM3_ESM.tif]
